# Effects of an herbal extract mixture (*Withania somnifera, Salvia rhytidea* Benth, *Perovskia abrotanoides*) on egg quality, carcass traits, gut health, immune response, and antioxidant status in laying Japanese quail (*Coturnix japonica*)

**DOI:** 10.1016/j.vas.2026.100617

**Published:** 2026-03-07

**Authors:** Mokhtar Ghajari, Hassan Saleh, Mohammad Taher Mirakzehi

**Affiliations:** a Department of Animal Science, Faculty of Agriculture, University of Saravan, Saravan, Sistan and Baluchestan, Iran; b Department of Animal Science, Faculty of Agriculture, University of Birjand, Birjand 97175-331, Iran

**Keywords:** Herbal extract mixture, Japanese quail, Egg quality, Gut health, Antioxidant status

## Abstract

Restrictions on antibiotic growth promoters have accelerated the search for sustainable phytogenic additives to support poultry productivity and health. This study evaluated the effects of an herbal extract mixture (HEM) containing *Withania somnifera, Salvia rhytidea*, and *Perovskia abrotanoides* on performance, egg quality, gut health, and physiological responses in laying Japanese quail (*Coturnix japonica*). A total of 600 eight‑week‑old quails were randomly assigned to five dietary treatments for 42 days: a negative control (basal diet), a positive control (basal diet supplemented with 40 mg/kg flumequine), and three experimental groups receiving the basal diet supplemented with 50, 100, or 150 mg/kg of the herbal extract mixture (HEM). The extract mixture was characterized for total phenolics and flavonoids using spectrophotometric assays. Supplementation with 100 mg kg⁻¹ HEM improved egg production and egg weight, yielding performance comparable to the positive control. In addition, dietary inclusion of 100 and 150 mg kg⁻¹ HEM was associated with improved yolk color, enhanced intestinal morphology, favorable modulation of ileal microbiota, and improved immune and antioxidant status, without adverse effects on feed efficiency or carcass traits. These findings indicate that HEM represents a promising phytogenic candidate for supporting health and productivity in laying Japanese quail.

## Introduction

The poultry industry faces increasing pressure to reduce antibiotic usage amid the global rise in antimicrobial resistance (AMR) and growing consumer demand for safe, antibiotic-free products (Surai et al., 2019; Hedman et al., 2020). This challenge has accelerated research into sustainable nutritional alternatives, particularly phytogenic feed additives, which include plant-derived compounds with antimicrobial, antioxidant, and immunomodulatory properties that can enhance poultry performance and health (Hashemipour et al., 2013; Alagawany et al., 2019; Sultan et al., 2024a, 2024b*;* Islam et al., 2024a*;* Aminullah et al*.,* 2025; Ali Tavakkoli, Mirakzehi, Saleh and Yousefi, 2021, Jafari, Saleh and Mirakzehi, 2021, Saleh, Mirakzehi and Jangjou, 2024).

Herbal extracts derived from *Withania somnifera* (WS) and *Salvia* species have attracted considerable attention as potential substitutes for antibiotic growth promoters. Previous studies have shown that WS can improve gut health, nutrient utilization, immune response, and oxidative status in poultry (Mirakzehi et al., 2017; Azimi et al., 2020; Salem et al., 2022). Similarly, *Salvia rhytidea* Benth. (SR) has been reported to exert antimicrobial and antioxidant activities that contribute to improved intestinal health and microbial balance (Salari et al., 2016; Saeghi et al., 2021; Bahadoran et al., 2023). *Perovskia abrotanoides* (PA), a medicinal plant adapted to arid environments, has also demonstrated antimicrobial and antioxidant potential, supporting its applicability in poultry feeding strategies (Ghafourian & Mazandarani, 2017; Saeghi et al., 2021)

However, despite the documented effects of WS, SR, and PA when used individually, their combined application in laying Japanese quail diets has not yet been systematically investigated. The present study therefore introduces a novel herbal extract mixture (HEM) composed of equal proportions of WS, SR, and PA collected from the hot and arid regions of Sistan and Baluchestan, Iran. Plants grown under such environmental conditions are often characterized by elevated concentrations of secondary metabolites, including phenolics and flavonoids, which may enhance their biological efficacy (Ghafourian and Mazandarani, 2017; Azizi et al., 2021; Sultan et al., 2024c; Islam et al., 2024b).

Japanese quail (*Coturnix japonica*) are widely used in nutritional and physiological research due to their rapid growth rate, early sexual maturity, and high egg production efficiency, making them a suitable experimental model for evaluating dietary phytogenic interventions (Abd El-Hack et al., 2016). Previous studies have demonstrated that phytogenic additives can positively influence egg quality, intestinal morphology, microbial populations, and immune responses in quail and other poultry species (Ghasemi et al., 2014; Lee et al., 2003).

It was hypothesized that dietary supplementation with this combined herbal extract mixture would improve production performance, gut health, immune response, and antioxidant status in laying Japanese quail compared with both antibiotic-supplemented and unsupplemented diets. Accordingly, the aim of the present study was to evaluate the effects of dietary HEM supplementation at levels of 50, 100, and 150 mg kg⁻¹, compared with a flumequine-supplemented diet (40 mg kg⁻¹) and an unsupplemented control, over a 42-day experimental period. The impacts of these treatments were assessed on egg quality, carcass traits, intestinal morphology, ileal microbial populations, hematological indices, immune responses, and antioxidant status in laying Japanese quail.

## Materials and methods

### Preparation and measurement of extracts and compounds

Roots of Withania somnifera (WS) and aerial parts (leaves and stems) of *Salvia rhytidea* Benth (SR) and Perovskia abrotanoides (PA) were collected from one-year-old cultivated plants grown in Saravan, Sistan and Baluchestan, Iran. Plant materials were dried, milled to a fine powder, and subjected to hydroalcoholic extraction. For each plant, 2.5 g of powdered material was extracted with 40 mL of 70% ethanol at room temperature for 48 h. The extracts were filtered under vacuum, concentrated using a rotary evaporator (Labrota 4000, Heidolph, Germany) at 30°C, and stored at –20°C (Saleh et al., 2018). The use of 70% ethanol as the extraction solvent was selected based on its proven efficiency in extracting a broad spectrum of polar and moderately non‑polar phytochemicals, including phenolics and flavonoids, while maintaining compound stability and minimizing degradation (Saleh et al., 2018).

The herbal extract mixture (HEM) was prepared by mixing equal proportions (1:1:1, w/w) of WS, SR, and PA extracts. To characterize the bioactive content of the extracts, analyses were performed for key phytochemical groups, as originally presented in Table 1 of the submitted manuscript. This table, which was included in the initial submission and is not a new addition, details the total polyphenol content (TPC) determined using the Folin–Ciocalteu method (Saleh et al., 2018), total flavonoid content (TFC) quantified via the aluminum chloride assay (Hassani Moghaddam & Shaaban, 2018), hydrolysable tannins (HTs) quantified in triplicate using potassium iodate (KIO₃) following Çam and Hısıl (2010), and condensed tannins (CTs) extracted with hydrochloric acid butanol and measured after heating at 100°C for 3 h based on absorbance at 550 nm (Saleh et al., 2018). All phytochemical analyses, including total phenolic content, total flavonoid content, hydrolysable tannins, and condensed tannins, were performed in triplicate, and results were expressed as mean values. Both TPC and TFC were measured with a UV–Vis spectrophotometer (Shimadzu, Japan). Key active compounds identified in the literature include withanolides (e.g., withaferin A) in WS (Mishra et al., 2008), diterpenoids such as salvigenin and rosmarinic acid in SR (Jamshidi-Kia et al., 2017), and tanshinones and abietane diterpenes in PA (Jamshidi-Kia et al., 2017).

**Table 1:** Some compound of extract the herbal extract mixture (HEM).

Item	
TPC (mgGAE/G DW)	208.9±0.85
TFC (mgQE/G DW)	105.48±1.3
Hydrolysable tannins, mg TAE /ml)	1.9±0.85
Condensed tannins, mg CE /ml)	0.02±1.3

(n=5) Values are expressed as means ±SD,

### Experimental design and diets

A total of 600 eight-week-old laying Japanese quail (*Coturnix japonica*), with an initial average body weight of 120 ± 5 g (mean ± SD), were used in this 42-day study. The birds were randomly allocated to one of five dietary treatment groups in a completely randomized design. Each treatment consisted of 12 replicate pens, with 10 birds per pen. The pen (cage) was considered the experimental unit for all performance and egg production parameters. The dietary treatments were: (1) a negative control (NC) consisting of a basal diet; (2) a positive control (PC), which was the basal diet supplemented with 40 mg/kg flumequine; and three experimental groups receiving the basal diet supplemented with 50 (HEM 50), 100 (HEM 100), or 150 (HEM 150) mg/kg of the herbal extract mixture (HEM).

The basal diet was a corn–soybean meal-based mash formulated to meet or exceed the nutrient requirements for laying quail as recommended by the NRC (1994), with its composition detailed in Table 2. The HEM was incorporated into the basal diets using a horizontal mixer to ensure uniform distribution. Throughout the experimental period, all birds were housed in battery cages (90 × 40 × 40 cm) under controlled environmental conditions (temperature: 22–24°C; relative humidity: 55–60%; photoperiod: 16L:8D) and had *ad libitum* access to feed and water

**Table 2:** Composition and nutrient levels of the basal diet for laying Japanese quail.

Ingredient	(%)	Calculated composition 2	
Yellow corn	58.70	Metabolizable energy, Kcal/kg	2900.00
Soybean meal 44%	27.50	Crude protein %	20.00
Corn gluten meal 62%	4.60	Calcium %	2.50
Vegetable oil	1.80	NonphytateP %	0.35
Limestone	5.54	Lysine %	1.00
Di-calciumphosphate	1.20	TSAA^3^ %	0.70
Salt	0.30		
Premix^1^	0.3		
L-Lysine%	0.06		
Dl-Methionine%	0.02		

1 Layer vitamin–mineral premix: Each 1 kg contains: vit. A, 8,000 IU; vit. D₃, 1,300 ICU; vit. E, 5 mg; vit. K, 2 mg; vit. B₁, 0.7 mg; vit. B₂, 3 mg; vit. B₆, 1.5 mg; vit. B₁₂, 7 mg; biotin, 0.1 mg; pantothenic acid, 6 g; niacin, 20 g; folic acid, 1 mg; manganese, 60 mg; zinc, 50 mg; copper, 6 mg; iodine, 1 mg; selenium, 0.5 mg; cobalt, 1 mg.

2 Calculated according to NRC (1994) ³, TSAA: Total sulfur amino acid.

### Sampling and measurements

#### Performance and egg quality

Daily records were kept for egg production (%), egg weight (g), egg mass (g/hen/day), feed intake (g/bird/day), and feed conversion ratio (FCR, g feed/g egg mass), following Zamanizadeh et al. (2021). Weekly, 20 eggs per treatment (two per replicate) were collected for quality assessment, including yolk percentage, yolk index (height/diameter), yolk colour (Roche colour fan), albumen percentage, Haugh unit, shell percentage, shell thickness (air cell, equator, sharp end; measured with a digital caliper, Mitutoyo, Japan), and shape index (width/length × 100) (Fikry et al., 2021). Each egg was used for a single quality assessment to avoid measurement interference among parameters.

### Carcass traits, gut morphology, and ileal microbiota

At the end of the trial, 20 quails per treatment (two per replicate) were weighed and euthanized via cervical dislocation. Carcass yield (%) and organ weights (breast, thigh, wing, back, liver, gizzard) were expressed as a percentage of live weight. Duodenum length was measured (Fikry et al., 2021). Ileal samples were fixed in 10% buffered formalin, paraffin-embedded, sectioned (5 μm), and stained with hematoxylin–eosin. Villus height, villus width, crypt depth, and villus height-to-crypt depth ratio were measured in 10 well-oriented villi per bird using a microscope (Olympus, Japan) equipped with a digital camera (Zamanizadeh et al., 2021). Histological measurements were performed by an observer blinded to the dietary treatments.

Ileal digesta were collected for microbiota analysis. Samples were serially diluted in sterile saline and plated on MRS agar (for *Lactobacillus* and *Bifidobacterium* spp.), reinforced clostridial agar (for *Clostridium* spp.), and MacConkey agar (for *Escherichia coli*). Plates were incubated anaerobically (*Lactobacillus, Bifidobacterium, Clostridium*) with GasPak jars or aerobically (*E. coli*) at 37°C for 24–48 h. Results were expressed as log₁₀ CFU/g (Fikry et al., 2021; Saleh et al., 2023).

### Blood biochemistry, immune indices, and antioxidant status

Blood samples were collected from the wing vein of 20 quail per treatment group (two birds per replicate) at the end of the experiment. Serum was isolated by centrifugation at 3000 × g for 10 min and then analyzed for lipid profiles, including high-density lipoprotein (HDL), low-density lipoprotein (LDL), triglycerides, and cholesterol (measured in mg/dL), as well as the hepatic enzymes alanine aminotransferase (ALT) and aspartate aminotransferase (AST) (measured in U/L). These analyses were performed using commercial kits from Pars Azmoon (Iran), following the manufacturer’s instructions. Immune parameters were assessed, including immunoglobulin levels (IgM, IgA, and IgY in mg/dL), percentages of heterophils and lymphocytes, and the heterophil-to-lymphocyte (H/L) ratio. These were determined using enzyme-linked immunosorbent assay (ELISA) and differential leukocyte counts from Giemsa-stained blood smears, as described by Saleh et al. (2023). Antioxidant status was evaluated by measuring serum glutathione peroxidase (GPx), catalase (CAT), and superoxide dismutase (SOD) (U/mL); total antioxidant capacity (TAC, mmol/L); and malondialdehyde (MDA, nmol/mL) using colorimetric assays (Fikry et al., 2021). The intra-assay coefficient of variation for antioxidant assays was <5%, according to the kit manufacturer.

### Statistical analysis

Data were analyzed using SAS 9.4 (SAS Institute, Cary, NC, USA). One-way ANOVA assessed treatment effects. Microbial counts and heterophil to lymphocyte ratio data were log₁₀ transformed prior to analysis to meet normality assumptions. The statistical model was:

$$Y_{ij}=\mu +T_{i}+\varepsilon_{ij}$$

where Yᵢⱼ = observation, μ = overall mean, Tᵢ = treatment effect, and εᵢⱼ = random error. Means were separated using Tukey’s test, with significance at *P* < 0.05.

## Results

### Egg quality and performance parameters

The effects of dietary treatments on performance and egg quality are summarized in Table 3, Table 4. Egg production and egg weight were significantly affected by dietary treatments, with the PC and HEM 100 groups showing superior performance compared with the NC and HEM 50 groups (*P* > 0.05). Regarding egg quality traits, dietary supplementation with HEM at 100 and 150 mg kg improved yolk color and eggshell percentage relative to the control diet. However, egg mass, feed intake, and feed conversion ratio were not affected by the dietary treatments**.**

**Table 3:** Effect of herbal extract mixture (HEM) on egg quality and performance in laying Japanese quail.

Treatment^1^	Egg Weight (g)	Egg Production (%)	Egg Mass (g)	Feed Intake (g/quail/d)	FCR
**NC**	11.20^b^	87.07^b^	10.33	28.11	2.724
**PC**	11.46^a^	90.03^a^	10.98	27.67	2.524
**Control +50 mg/kg HEM**	11.25^b^	87.85^b^	10.27	27.93	2.714
**Control +100 mg/kg HEM**^1^	11.35^a^	89.32^a^	10.83	27.24	2.514
**Control +150 mg/kg HEM**^1^	11.35^ab^	89.92^a^	10.74	27.24	2.54
**SEM**^2^	0.097	5.132	0.631	0.115	0.035
**P-value**	0.001	0.001	0.348	0.248	0.109

1 Treatments consisted of: NC (Negative Control; basal diet); PC (Positive Control; basal diet + 40 mg/kg flumequine); and HEM 50, 100, 150 (basal diet supplemented with 50, 100, or 150 mg/kg of the herbal extract mixture, respectively). The herbal extract mixture (HEM) was composed of equal parts of *Withania somnifera* (root), *Salvia rhytidea*, and *Perovskia abrotanoides* (aerial parts)

2 SEM = Standard Error of the Means (n = 12 replicates per treatment). ^a–d^ Means within a column lacking a common superscript letter differ significantly (*P* < 0.05).

**Table 4:** Effect of herbal extract mixture (HEM) on egg quality traits in laying Japanese quail.

Treatment^1^	Yolk (%)	Yolk Index	Yolk Color	Albumen (%)	Haugh Unit	Shell (%)	Shell Thickness (mm)	Shape Index
**NC**	31.59	38.68	5.02^b^	58.56	85.36	9.65^b^	0.22	76.07
**PC**	31.37	38.79	5.00^b^	59.02	85.42	9.61^b^	0.22	76.87
**Control +50 mg/kg HEM**	30.92	39.28	5.75^ab^	59.37	85.43	9.70^ab^	0.23	76.11
**Control +100 mg/kg HEM**	30.33	39.32	6.02^a^	59.71	85.79	9.96^a^	0.24	76.66
**Control +150 mg/kg HEM**	30.15	39.22	6.13^a^	59.86	86.17	9.98^a^	0.24	75.38
**SEM**^2^	0.358	0.835	0.042	0.168	0.294	0.014	0.001	0.718
**P-value**	0.348	0.358	0.035	0.458	0.635	0.041	0.194	0.561

1 Treatments consisted of: NC (Negative Control; basal diet); PC (Positive Control; basal diet + 40 mg/kg flumequine); and HEM 50, 100, 150 (basal diet supplemented with 50, 100, or 150 mg/kg of the herbal extract mixture, respectively). The herbal extract mixture (HEM) was composed of equal parts of *Withania somnifera* (root), *Salvia rhytidea*, and *Perovskia abrotanoides* (aerial parts)

2 SEM = Standard Error of the Means (n = 12 replicates per treatment). ^a–d^ Means within a column lacking a common superscript letter differ significantly (*P* < 0.05).

### Carcass traits

As shown in Table 5, the dietary treatments had no significant effect on any of the measured carcass characteristics. The relative weights of the carcass, breast, thigh, internal organs (liver, gizzard), and the length of the duodenum did not differ among the experimental groups (*P* > 0.05).

**Table 5:** Effect of herbal extract mixture (HEM) on carcass traits in laying Japanese quail.

Treatment^1^	Carcass (%)	Breast (%)	Thigh (%)	Wing (%)	Back (%)	Liver (%)	Gizzard (%)	Duodenum Length (cm)
**NC**	75.23	22.12	14.18	4.24	9.20	2.66	1.05	13.70
**PC**	75.76	22.68	14.68	4.65	9.64	2.45	1.06	13.60
**Control +50 mg/kg HEM**	75.43	22.31	14.49	4.88	9.51	2.62	1.05	13.86
**Control +100 mg/kg HEM**	75.75	22.42	14.62	4.58	9.70	2.53	1.04	14.05
**Control +150 mg/kg HEM**	75.76	22.49	14.72	4.82	9.28	2.50	1.06	13.58
**SEM**^2^	2.453	0.436	0.333	0.129	0.424	0.189	0.036	0.351
**P-value**	0.578	0.245	0.365	0.137	0.562	0.429	0.537	0.687

1 Treatments consisted of: NC (Negative Control; basal diet); PC (Positive Control; basal diet + 40 mg/kg flumequine); and HEM 50, 100, 150 (basal diet supplemented with 50, 100, or 150 mg/kg of the herbal extract mixture, respectively). The herbal extract mixture (HEM) was composed of equal parts of *Withania somnifera* (root), *Salvia rhytidea*, and *Perovskia abrotanoides* (aerial parts)

2 SEM = Standard Error of the Means (n = 12 replicates per treatment). ^a–d^ Means within a column lacking a common superscript letter differ significantly (*P* < 0.05).

### Ileal morphology

Dietary treatments significantly influenced ileal morphology (Table 6). Birds fed diets supplemented with 100 and 150 mg/kg HEM exhibited significantly greater villus height compared to the NC group (*P* <0.05). Conversely, crypt depth was significantly reduced in the PC, HEM 100, and HEM 150 groups compared to the NC group (*P <*0.05). Villus width and the villus height-to-crypt depth ratio were not significantly affected by the treatments (*P* > 0.05).

**Table 6:** Effect of herbal extract mixture (HEM) on gut morphology in laying Japanese quail.

Treatment^1^	Villus Height (µm)	Villus Width (µm)	Crypt Depth (µm)	Villus Index
**NC**	625.24^b^	88.32	124.22^a^	6.17
**PC**	661.23^ab^	89.96	104.24^c^	5.31
**Control +50 mg/kg HEM**	655.23^ab^	89.12	111.26^ab^	5.24
**Control +100 mg/kg HEM**	675.23^a^	90.90	101.01^c^	5.57
**Control +150 mg/kg HEM**	689.23^a^	89.89	99.26^c^	5.79
**SEM**^2^	27.753	5.321	4.325	0.228
**P-value**	0.021	0.543	0.001	0.568

1 Treatments consisted of: NC (Negative Control; basal diet); PC (Positive Control; basal diet + 40 mg/kg flumequine); and HEM 50, 100, 150 (basal diet supplemented with 50, 100, or 150 mg/kg of the herbal extract mixture, respectively). The herbal extract mixture (HEM) was composed of equal parts of *Withania somnifera* (root), *Salvia rhytidea*, and *Perovskia abrotanoides* (aerial parts)

2 SEM = Standard Error of the Means (n = 12 replicates per treatment). ^a–d^ Means within a column lacking a common superscript letter differ significantly (*P* < 0.05).

### Ileal microbiota population

Dietary treatments were associated with differences in ileal microbial counts (Table 7). Lactobacillus populations were higher in the HEM 100 and HEM 150 groups compared with the PC group (P < 0.05). *Clostridium* spp. and *Escherichia coli* counts were lower in the PC, HEM 100, and HEM 150 groups than in the NC group. Bifidobacterium counts did not differ among dietary treatments.

**Table 7:** Effect of herbal extract mixture (HEM) on gut microbial population in laying Japanese quail (Log10 cfu/g).

Treatment^1^	*Lactobacillus*	*Bifidobacterium*	*Clostridium*	*E. coli*
**NC**	6.64^b^	5.53	7.23^a^	5.58^a^
**PC**	5.03^c^	4.42	4.63^cd^	3.57^c^
**Control +50 mg/kg HEM**	7.15^ab^	5.62	6.56^ab^	4.91^b^
**Control +100 mg/kg HEM**	7.88^a^	5.68	5.23^c^	3.82^c^
**Control +150 mg/kg HEM**	7.75^a^	5.63	5.13^c^	3.84^c^
**SEM**^2^	0.519	0.365	0.382	0.315
**P-value**	0.001	0.091	0.001	0.001

1 Treatments consisted of: NC (Negative Control; basal diet); PC (Positive Control; basal diet + 40 mg/kg flumequine); and HEM 50, 100, 150 (basal diet supplemented with 50, 100, or 150 mg/kg of the herbal extract mixture, respectively). The herbal extract mixture (HEM) was composed of equal parts of *Withania somnifera* (root), *Salvia rhytidea*, and *Perovskia abrotanoides* (aerial parts)

2 SEM = Standard Error of the Means (n = 12 replicates per treatment). ^a–d^ Means within a column lacking a common superscript letter differ significantly (*P* < 0.05).

### Blood biochemistry

The effects of dietary treatments on blood biochemical parameters are presented in Table 8. Supplementing the diet with HEM at all levels (50, 100, and 150 mg/kg) significantly reduced the serum activities of alanine aminotransferase (ALT) and aspartate aminotransferase (AST) when compared with the PC group (*P*<0.05). However, serum concentrations of HDL, LDL, triglycerides, and total cholesterol were not significantly affected by the dietary treatments (*P*>0.05).

**Table 8:** Effect of herbal extract mixture (HEM) on blood parameters and hepatic enzymes in laying Japanese quail.

Treatment^1^	HDL^2^ (mg/dL)	LDL (mg/dL)	Triglycerides (mg/dL)	Cholesterol (mg/dL)	ALT (U/L)	AST (U/L)
**NC**	50.27	138.27	283.05	229.49	13.25^b^	182.23^b^
**PC**	56.53	147.50	311.25	239.20	14.63^a^	193.26^a^
**Control +50 mg/kg HEM**	46.47	133.50	262.03	204.17	12.35^c^	167.68^c^
**Control +100 mg/kg HEM**	47.54	123.49	234.17	198.14	12.02^c^	164.46^c^
**Control +150 mg/kg HEM**	46.14	119.88	239.11	202.54	12.12^c^	178.08^c^
**SEM**^3^	2.548	5.223	8.035	6.290	0.081	3.328
**P-value**	0.113	0.083	0.116	0.073	0.001	0.001

1 Treatments consisted of: NC (Negative Control; basal diet); PC (Positive Control; basal diet + 40 mg/kg flumequine); and HEM 50, 100, 150 (basal diet supplemented with 50, 100, or 150 mg/kg of the herbal extract mixture, respectively). The herbal extract mixture (HEM) was composed of equal parts of *Withania somnifera* (root), *Salvia rhytidea*, and *Perovskia abrotanoides* (aerial parts).

2 HDL: High-Density Lipoprotein; LDL: Low-Density Lipoprotein; ALT: Alanine Transaminase; AST: Aspartate Transaminase.

3 SEM = Standard Error of the Means (n = 12 replicates per treatment). ^a–d^ Means within a column lacking a common superscript letter differ significantly (*P* < 0.05).

### Immune parameters

As shown in Table 9, dietary HEM supplementation influenced several immune-related parameters. IgA and IgY concentrations were higher in the HEM-supplemented groups compared with the NC and PC groups (P < 0.05). The heterophil to lymphocyte (H/L) ratio differed among treatments. IgM concentrations were not affected by dietary treatments.

**Table 9:** Effect of herbal extract mixture (HEM) on immune parameters in laying Japanese quail.

Treatment^1^	IgM^2^ (mg/dL)	IgA (mg/dL)	IgY (mg/dL)	Heterophil (%)	Lymphocyte (%)	H/L Ratio
**NC**	0.58	0.24^b^	0.75^b^	45.50	55.50	0.83^a^
**PC**	0.57	0.25^b^	0.74^b^	43.50	55.50	0.78^ab^
**Control +50 mg/kg HEM**^1^	0.607	0.285^a^	0.78^a^	41.50	58.50	0.71^b^
**Control +100 mg/kg HEM**	0.617	0.23^a^	0.790^a^	38.50	61.50	0.63^bc^
**Control +150 mg/kg HEM**	0.601	0.29^a^	0.795^a^	38.000	62.000	0.62^bc^
**SEM**^2^	0.001	0.001	0.004	0.080	0.080	0.013
**P-value**	0.034	0.015	0.043	1.783	1.783	0.044

1 Treatments consisted of: NC (Negative Control; basal diet); PC (Positive Control; basal diet + 40 mg/kg flumequine); and HEM 50, 100, 150 (basal diet supplemented with 50, 100, or 150 mg/kg of the herbal extract mixture, respectively). The herbal extract mixture (HEM) was composed of equal parts of *Withania somnifera* (root), *Salvia rhytidea*, and *Perovskia abrotanoides* (aerial parts).

2 IgM: Immunoglobulin M; IgA: Immunoglobulin A; IgY: Immunoglobulin Y. H/L Ratio: Heterophil /Lymphocyte ^3^ SEM = Standard Error of the Means (n = 12 replicates per treatment). a–d Means within a column lacking a common superscript letter differ significantly (*P* < 0.05).

### Antioxidant status

Antioxidant‑related parameters are summarized in Table 10. Birds receiving 100 and 150 mg kg HEM exhibited higher activities of glutathione peroxidase (GPx) and superoxide dismutase (SOD) compared with the control groups (P < 0.05). Serum malondialdehyde (MDA) concentrations were lower in HEM‑supplemented groups. Catalase (CAT) activity did not differ among treatments.

**Table 10:** Effect of herbal extract mixture (HEM) on antioxidant status in laying Japanese quail.

Treatment^1^	GPX 2 (U/mL)	CAT (U/mL)	SOD (U/mL)	TAC (nmol/mL)	MDA (nmol/mL)
**NC**	893.220^c^	14.950	52.655^c^	3.653	3.731^a^
**PC**	899.200^c^	14.870	53.386^c^	3.723	3.615^a^
**Control +50 mg/kg HEM**	928.380^b^	15.660	56.140^ab^	5.345	2.915^b^
**Control +100 mg/kg HEM**	1035.175^a^	15.970	58.950^a^	7.635	2.465^c^
**Control +150 mg/kg HEM**	968.235^ab^	15.655	58.845^a^	7.421	2.620^bc^
**SEM**^3^	29.257	0.513	1.351	0.109	0.083
**P-value**	0.001	0.193	0.012	0.235	0.001

1 Treatments consisted of: NC (Negative Control; basal diet); PC (Positive Control; basal diet + 40 mg/kg flumequine); and HEM 50, 100, 150 (basal diet supplemented with 50, 100, or 150 mg/kg of the herbal extract mixture, respectively). The herbal extract mixture (HEM) was composed of equal parts of *Withania somnifera* (root), *Salvia rhytidea*, and *Perovskia abrotanoides* (aerial parts).

2 GPx: Glutathione peroxidase; CAT: Catalase; SOD: Superoxide dismutase; TAC:Total antioxidant capacity; MDA:Malondialdehyde;

3 SEM = Standard Error of the Means (n = 12 replicates per treatment). a–d Means within a column lacking a common superscript letter differ significantly (*P* < 0.05).

## Discussion

The effects observed in the present study may be related to the bioactive phytochemical constituents of herbal extract mixture (HEM), particularly phenolic compounds and flavonoids, which are known to exert antioxidant and biological activities.

The HEM, a combination of ethanolic extracts from the roots of *Withania somnifera* and the aerial parts of *Salvia rhytidea* and *Perovskia abrotanoides*, possessed a rich phytochemical profile that underpins its diverse biological activities (Table 1). The high total phenolic (TPC; 208.9 ± 0.85 mg GAE/g) and flavonoid content (TFC; 105.48 ± 1.3 mg QE/g) reflect substantial phytochemical richness and suggest strong potential for synergistic bioactivity. Crucially, the phytochemical balance appears optimised for efficacy; the mixture contained a notable level of hydrolysable tannins (1.9 ± 0.85 mg TAE/g), contributing to its antimicrobial properties, while the very low content of condensed tannins (0.02 ± 0.013 mg CE/g) minimises the risk of anti-nutritional effects, such as impaired protein and mineral absorption (Fernando et al., 2013; Gessner et al., 2017).

The high TPC and TFC values of the HEM are consistent with, and in some cases exceed, levels reported for its individual plant components, highlighting the potential for additive or synergistic effects. For instance, the phytochemical load is heavily influenced by *W. somnifera*, whose root extracts are known to yield high concentrations of withanolides and phenolic acids responsible for its documented antioxidant, immunomodulatory, and adaptogenic effects (Polumackanycz et al., 2023; Singirala et al., 2025). Similarly, *S. rhytidea* contributes significantly with compounds like rosmarinic acid, which confers not only antimicrobial activity but also modulates epithelial barrier integrity (Fallah Zahabi et al., 2020; Azizi et al., 2021). The inclusion of *P. abrotanoides*, a source of potent radical-scavenging glycosides, further strengthens the mixture’s overall antioxidant profile (Ghafourian & Mazandarani, 2017). Future investigations using GC–MS analysis may offer additional insights into the specific phytochemical constituents of HEM and their potential roles in the observed effects.

The robust phytochemical profile of HEM was associated with improvements in production performance. Supplementation with 100 mg/kg HEM enhanced egg production and egg weight to levels statistically comparable to the positive control (flumequine), suggesting that HEM may support reproductive output through distinct biological pathways. The adaptogenic properties of *W. somnifera*, mediated by withanolides, likely improved metabolic efficiency and resilience to physiological stress, thereby promoting greater resource allocation towards egg production (Azimi et al., 2020; Salem et al., 2022). Furthermore, HEM positively influenced key egg quality traits. The enhanced yolk pigmentation in birds receiving 100 and 150 mg/kg HEM is attributable to the deposition of dietary flavonoids and polyphenols into the yolk lipoproteins, a phenomenon reported for other phytogenic additives (Hassani Moghaddam & Shaaban, 2018; Balčiūnaitienė et al., 2022). Similarly, the increased eggshell percentage points towards improved calcium metabolism, a benefit potentially stemming from enhanced intestinal mineral absorption efficiency or direct modulation of Ca²⁺ transporters by *W. somnifera* bioactives (Mirakzehi et al., 2017; Saleh et al., 2024). The absence of effects on other egg quality parameters suggests a targeted mechanism of action, a pattern also observed with *Thymus vulgaris* supplementation (Ghasemi et al., 2014).

While HEM supplementation did not alter carcass characteristics—a finding consistent with phytogenic additives whose primary impacts are often functional rather than structural (Yu et al., 2025)—it was associated with marked improvements in gut health. Significantly, HEM enhanced intestinal morphology by increasing villus height and reducing crypt depth, with effects comparable or superior to the positive control. These architectural improvements are may be associated with HEM’s ability to modulate the gut microbial ecosystem. In this regard, HEM supplementation was accompanied by a beneficial shift in the intestinal microbiota, characterized by increased *Lactobacillus* populations and reduced counts of pathogenic *Clostridium* and *E. coli*. Such selective antimicrobial effects have been reported for polyphenols and tannins present in phytogenic additives, which may interfere with pathogenic bacterial membranes and metabolic processes while exerting limited effects on beneficial species (Salari et al., 2016; Saeghi et al., 2021).

The systemic benefits of improved gut health were further evidenced by the serum biochemistry and immune parameters. The observed reductions in ALT and AST levels across all HEM groups suggest a significant hepatoprotective effect, likely stemming from two synergistic mechanisms: reduced endotoxin translocation across the fortified gut barrier and the direct antioxidant action of HEM’s polyphenols on hepatocytes (Aljumaah et al., 2020a, 2020b; Azimi et al., 2020; Balčiūnaitienė et al., 2022). While HEM did not alter lipid profiles, indicating a targeted rather than broad metabolic impact, it was associated with enhanced humoral immunity. The elevated IgA and IgY titres in HEM-supplemented birds, which surpassed those of the positive control, point to potent immunomodulatory activity. This effect is likely driven by flavonoids and withanolides known to promote lymphocyte proliferation and antibody production (Mohiti-Asli & Ghanaatparast-Rashti, 2017; Kumari et al., 2020). Concurrently, the significant reduction in the heterophil-to-lymphocyte (H/L) ratio serves as a clear physiological indicator of reduced stress, suggesting a lower physiological stress status in HEM‑supplemented birds, which is consistent with the reported adaptogenic properties of W. somnifera; however, direct modulation of the HPA axis was not evaluated in the present study.

Finally, HEM supplementation was associated with enhancements in the birds’ endogenous antioxidant defense system. The increased activities of key antioxidant enzymes, including glutathione peroxidase (GPX) and superoxide dismutase (SOD), along with reduced malondialdehyde (MDA) levels, indicate an improved oxidative status and reduced lipid peroxidation. These findings suggest that HEM bioactive compounds, particularly phenolic acids and flavonoids, may influence antioxidant defense mechanisms beyond direct radical scavenging.

It can be hypothesized that such effects are related to the modulation of cellular antioxidant pathways, potentially involving Nrf2‑dependent signaling; however, this pathway was not directly assessed in the present study. The selective enhancement of specific antioxidant enzymes, rather than a generalized increase in total antioxidant capacity (TAC), may explain the lack of significant effects on TAC and catalase (CAT) activity. Similar patterns of antioxidant enzyme modulation have been reported for other phytogenic additives, such as Nigella sativa (Abd El‑Hack et al., 2016; Singirala et al., 2025), supporting the plausibility of this hypothesis.

In conclusion, dietary supplementation with the herbal extract mixture (HEM) positively influenced several health‑related parameters in laying Japanese quail. Inclusion of HEM at 100–150 mg/kg improved egg production and egg weight, enhanced egg quality traits, and was associated with better gut morphology, favorable modulation of ileal microbiota, improved immune responses, and enhanced antioxidant status. However, feed conversion ratio and egg mass were not significantly affected by dietary treatments. These findings suggest that HEM may serve as a promising phytogenic additive to support health and selected productive traits in laying quail, contributing to antibiotic‑reduction strategies in poultry production.

## Ethics statement

The protocol for this study received approval from the Research Animal Ethics Committee at the University of Saravan and complied with the standards established by the Iranian Council of Animal Care. All experimental procedures were carried out in accordance with the ARRIVE guidelines and the National Institutes of Health (NIH) standards for animal research (Percie du Sert et al., 2020).

## Ethical statement

All experimental procedures were reviewed and approved by the Research Animal Ethics Committee of the University of Saravan, adhering to the guidelines of the Iranian Council of Animal Care. The study was conducted in accordance with the ARRIVE guidelines (du Sert et al., 2020) and the National Institutes of Health (NIH) standards (Publication No. 85-23, revised 2011) for the care and use of laboratory animals, ensuring all efforts were made to minimise animal

## Funding statement

This research was supported by the University of Saravan. The funding body had no role in the design of the study, data collection and analysis, interpretation of results, or preparation of the manuscript.

## Data availability statement

All data used in this research are presented within the main article and its accompanying supplementary files.

## CRediT authorship contribution statement

**Mokhtar Ghajari:** Methodology, Funding acquisition, Formal analysis, Data curation, Writing – original draft, Writing – review & editing. **Hassan Saleh:** Writing – review & editing, Writing – original draft, Supervision, Conceptualization. **Mohammad Taher Mirakzehi:** Writing – review & editing, Validation, Software, Project administration.

## Declaration of competing interest

The authors declare no conflict of interest in conducting this study. The funding sources had no involvement in the design of the study, collection, analysis, interpretation of data, writing of the report, or in the decision to submit the article for publication.
